# Genome-wide identification and expression analysis of the glutamate receptor gene family in sweet potato and its two diploid relatives

**DOI:** 10.3389/fpls.2023.1255805

**Published:** 2023-12-21

**Authors:** Yaya Hu, Zhuoru Dai, Jinan Huang, Meikun Han, Zhiwei Wang, Weijing Jiao, Zhiyuan Gao, Xinliang Liu, Lanfu Liu, Zhimin Ma

**Affiliations:** ^1^ Hebei Key Laboratory of Crop Genetics and Breeding, Institute of Cereal and Oil Crops, Hebei Academy of Agriculture and Forestry Sciences, Shijiazhuang, Hebei, China; ^2^ Key Laboratory of Sweet Potato Biology and Biotechnology, Ministry of Agriculture and Rural Affairs, College of Agronomy & Biotechnology, China Agricultural University, Beijing, China; ^3^ Department of Agriculture Forestry and Biological Engineering, Baoding Vocational and Technical College, Baoding, Hebei, China; ^4^ School of Life Sciences, Jiangsu Normal University, Xuzhou, Jiangsu, China

**Keywords:** glutamate receptor, tissue-specific expression, root rot stress, abiotic stress, sweet potato, *Ipomoea trifida*, *Ipomoea triloba*

## Abstract

Plant glutamate receptor (GLR) homologs are crucial calcium channels that play an important role in plant development, signal transduction, and response to biotic and abiotic stresses. However, the *GLR* gene family has not yet been thoroughly and systematically studied in sweet potato. In this study, a total of 37 *GLR* genes were identified in the cultivated hexaploid sweet potato (*Ipomoea batatas*), and 32 *GLR* genes were discovered in each of the two diploid relatives (*Ipomoea trifida* and *Ipomoea triloba*) for the first time. Based on their evolutionary relationships to those of *Arabidopsis*, these *GLRs* were split into five subgroups. We then conducted comprehensive analysis to explore their physiological properties, protein interaction networks, promoter *cis*-elements, chromosomal placement, gene structure, and expression patterns. The results indicate that the homologous *GLRs* of the cultivated hexaploid sweet potato and its two relatives are different. These variations are reflected in their functions related to plant growth, hormonal crosstalk, development of tuberous roots, resistance to root rot, and responses to abiotic stress factors, all of which are governed by specific individual *GLR* genes. This study offers a comprehensive analysis of *GLR* genes in sweet potato and its two diploid relatives. It also provides a theoretical basis for future research into their regulatory mechanisms, significantly influencing the field of molecular breeding in sweet potatoes.

## 1 Introduction

Glutamate, a ubiquitous amino acid, participates in various important chemical reactions in animals, plants, and microorganisms and plays an indispensable function in protein metabolism and signal transduction processes (Forde and Lea, 2007). Glutamate receptors (GLRs), including the ionotropic and metabotropic GLRs, were first discovered in animals. The ionotropic GLR of animal is a ligand-gated non-selective cation channel that regulates excitatory nerve signal transmission and guides neuronal development (Mayer, 2006; Zhu and Gouaux, 2017). Due to the existence of conserved signature domains, plant GLR structures are similar to those for animal ionotropic GLRs (Wudick et al., 2018a). Plant GLRs composed of receptor domains and four transmembrane helical domains are typical membrane proteins belonging to a class of amino acid-gated Ca^2+^ channels. When GLRs are activated by their corresponding ligands, they can mediate the transmembrane influx of Ca^2+^, thus activating Ca^2+^ signaling to regulate plant responses to stress and simultaneously impact their overall development and growth (Sobolevsky et al., 2009; Traynelis et al., 2010; Kong et al., 2015; Tian et al., 2020; Luan and Wang, 2021; Ahmed et al., 2023).

Plant GLRs have been studied for more than 25 years, during which significant progress has been made in understanding their structural and functional characteristics. At present, 20, 13, 24, 34, 29, 35, 36, 16, 43, 16, and 21 *GLRs*, have been identified in *Arabidopsis thaliana* (Chiu et al., 2002), *Solanum lycopersicum* (Aouini et al., 2012), *Oryza sativa* (Singh et al., 2014), *Pyrus bretschneideri* (Chen et al., 2016), *Medicago truncatula* (Philippe et al., 2019), *Glycine max* (Zeng et al., 2020), *Gossypium hirsutum* (Liu et al., 2021), *Zea mays* (Zhou et al., 2021), *Saccharum* (Zhang et al., 2022), *Brassica rapa* (Yang et al., 2022), and *Erigeron breviscapus* (Yan et al., 2022), respectively. Furthermore, genetic research has demonstrated that *GLRs* play a crucial role in regulating various plant developmental processes and responding to environmental stresses such as salt, drought, heat, wounding, and pathogen attacks. These developmental processes encompass seed germination (Kong et al., 2015), root development (Vincill et al., 2013; Singh et al., 2016), hypocotyl elongation and pollen tube growth (Michard et al., 2011; Wudick et al., 2018b), xylem growth (Chen et al., 2016), Ca^2+^ distribution (Kim et al., 2001), stomatal closure (Cho et al., 2009), nitrogen and carbon metabolism (Kang et al., 2004), and abscisic acid (ABA) synthesis. Tea transcriptome data showed that the homologs for *GLR2.7* and *GLR2.8* manifest an upregulation in their expression levels under salt stress (Wan et al., 2018). Following coldstress, *AtGLR1.2* and *AtGLR1.3* activate core binding factors*/*dehydration-responsive element binding protein 1 signaling pathway through endogenous jasmonic acid (JA) accumulation, which contributes towards enhancing cold tolerance (Hu et al., 2013; Zheng et al., 2018). Moreover, *OsGLR3.4* is involved in brassinosteroid-mediated damage response from root to stem in rice. (Yu et al., 2022). GLRs can also improve the regeneration of plants after wounding and are crucial for establishing a balance between plant defense and regeneration following injury (Bian et al., 2022; Hernández-Coronado et al., 2022). As Ca^2+^ channels, plant GLRs can participate in disease-resistance responses by regulating Ca^2+^ signals. Kang et al. cloned an *RsGLuR* gene located on the plasma membrane from small radish, and found that overexpression of *RsGLuR* in *Arabidopsis* can improve resistance to the pathogen *Botrytis cinema* by triggering JA biosynthesis (Kang et al., 2006). However, there is no substantial volume of research on *GLRs* in sweet potato, in terms of their regulatory mechanisms and biological functions.

Sweet potato, *Ipomoea batatas* (L.) Lam. (2n = 6x = 90), is characterized by drought resistance, high and stable yields, strong adaptability, and rich nutrition, and is the best food recommended by the World Health Organization (Ma et al., 2012). It is both a food and cash crop with a wide range of uses. Specifically, it can be used as fresh food (Xie et al., 2018), starch processing (Zhou et al., 2020), food processing (Xiang et al., 2020), leafy vegetables (Su et al., 2018), and ornamental purposes (Meng and Lai, 2019). Accordingly, sweet potato has become an essential feed, food, and industrial raw material, and it is widely cultivated in over 100 regions and countries across the globe (Food and Agriculture Organization of the United Nations, 2021). The sweet potato genome is large and complex owing to its hexaploidy and high heterozygosity. In recent years, the assembly and reporting of the genomes for hexaploid sweet potato (Taizhong6) and its two diploid relatives (*Ipomoea trifida*, NCNSP0306, 2n=2x=30 and *Ipomoea triloba*, NCNSP0323, 2n=2x=30) have made it possible to analyze and identify the important gene family in sweet potato across the whole-genome level (Yang et al., 2017; Wu et al., 2018).

The cultivated hexaploid sweet potato and two of its diploid relatives (*I. trifida* and *I. triloba*) were used to screen and identify the *GLR* gene members in this study. The *GLR* genes were then analyzed for phylogenetic relationships, protein physicochemical properties, chromosomal localizations, gene structures, promoter *cis*-elements, protein interaction network, and expression patterns. The findings provide a basis for further understanding of the biological functions of *GLRs* and the future molecular breeding of sweet potatoes.

## 2 Materials and methods

### 2.1 Identification of *GLRs*

The whole-genome sequences for *I. batatas*, *I. trifida*, and *I. triloba* were obtained through *Ipomoea* Genome Hub (https://ipomoea-genome.org/) (viewed: 6 March 2023) and Sweetpotato Genomics Resource (http://sweetpotato.plantbiology.msu.edu/) (viewed: 6 March 2023). Three different screening techniques (Dai et al., 2022) were used at once to ensure that all members of *GLR* family were accurately identified.

### 2.2 Prediction for GLR protein properties

The molecular weight, hydrophilicity, instability index, and theoretical isoelectric point for GLRs were computed through the Expert Protein Analysis System (ExPASy, https://www.expasy.org/) (viewed: 12 March 2023). Protein Subcellular Localization Prediction (PSORT, https://wolfpsort.hgc.jp/) (viewed: 12 March 2023) was employed to predict the subcellular localizations for GLRs.

### 2.3 Chromosomal distribution for *GLRs*

*IbGLRs*, *ItfGLRs*, and *ItbGLRs* were separately mapped to the respective chromosomes for *I. batatas*, *I. trifida*, and *I. triloba* by *Ipomoea* Genome Hub (https://ipomoea-genome.org/) (viewed: 14 March 2023) and Sweetpotato Genomics Resource (http://sweetpotato.plantbiology.msu.edu/) (viewed: 14 March 2023). The Toolkit for Biologists integrating various biological data handling tools (TBtools) program (South China Agricultural University, Guangzhou, China) was used for visualization.

### 2.4 Phylogenetic analysis for *GLRs*

MEGA ClustalW 7.0 was used to phylogenetically analyze the amino acid sequences for GLRs of *Arabidopsis*, *I. batatas*, *I. trifida*, and *I. triloba* (Kumar et al., 2016). Bootstrapping was carried out with 1000 replicates, and Interactive Tree of Life (iTOL) software (http://itol.embl.de/) (viewed: 8 June 2023) was used to construct a phylogenetic tree.

### 2.5 Domain identification and conserved motif analysis for GLRs

To perform an in-depth study of GLRs’ conserved motifs, the Multiple Expectation Maximizations for Motif Elicitation (MEME) program (https://meme-suite.org/meme/) (viewed: 15 March 2023) was used. The maximum number of motifs that could be used has been set at five.

### 2.6 Exon-intron structures and promoter analysis for *GLRs*

A database of Plant Cis-Acting Regulatory Element (PlantCARE, (http://bioinformatics.psb.ugent.be/webtools/plantcare/html) (viewed: 16 March 2023) predicted *cis*-elements within 1500 bp promoter region for *GLRs* (Lescot et al., 2002). Gene Structure Display Server (GSDS) 2.0 developed by Peking University, Beijing, China (http://gsds.gao-lab.org/) (viewed: 16 March 2023) and the TBtools software developed by South China Agricultural University, Guangzhou, China was used to obtain and visualize the exon-intron structures for *GLRs*, respectively.

### 2.7 GLR protein interaction networking

Depending upon *Arabidopsis* orthologous proteins, the Search Tool for the Retrieval of Interacting Genes/Proteins (STRING, https://www.string-db.org/) (viewed: 18 March 2023) predicted GLR protein interaction network. The Cytoscape version 3.2 was used to construct the network map (Kohl et al., 2011).

### 2.8 Data analysis

Transcriptome analysis and real-time quantitative PCR (qRT-PCR) was carried out at duration points (0 h, 36 h, 72 h, 120 h, and 10 d) following root rot induction using the underground stem of resistant variety Jishuzi203 and susceptible variety Jishuzi563. These plants were provided by the Institute of Cereal and Oil Crops, Hebei Academy of Agriculture and Forestry Sciences (Shijiazhuang, China) and planted in the natural root rot disease nursery in Xiong'an New Area, China. qRT-PCR was conducted on a CFX Opus 384 Real-Time PCR system (Bio-Rad, USA) by the ChamQ Universal SYBR qPCR Master Mix (Vazyme, China). The relative expression level of the target gene was presented as fold change compared with the internal control using the 2 ^- Δct^ method, and data were analyzed with Duncan’s multiple range test (p < 0.05). Three biological replications were performed for each test. A gene of *I. batatas* ADP-ribosylation factor (ARF, GenBank JX177359) was used as an internal control. The specific primers used for the qRT-PCR analysis were listed in **Supplementary Table S2**. Based on related investigations (Zhang et al., 2017; Dong et al., 2019; Zhu et al., 2019) ribonucleic nucleic acid sequencing (RNA-seq) data for *IbGLRs* were collected. RNA-seq data for *ItfGLRs* and *ItbGLRs* were collected through Sweetpotato Genomics Resource (http://sweetpotato.plantbiology.msu.edu/) (viewed: 22 March 2023). The fragments per kilobase of exon per million mapped fragments (FPKM) method was used to calculate the *GLRs* expression levels. Tbtools was used to build the heat maps.

## 3 Results

### 3.1 Characterization of *GLRs* in sweet potato and two diploid relatives

37 *GLRs* for sweet potato (named *Ib*) and 32 for each of two diploid relatives (named *Itf* and *Itb*) were identified through a combination of three methods used by Dai et al. (2022). The sequences from *I. batatas* were used for the analysis of the physicochemical features of *GLRs* (**Table 1**). The coding sequence length of *IbGLRs* varied from 1977 bp (*IbGLR7*) to 7326 bp (*IbGLR36*). The molecular weights of IbGLRs ranged from 73.627 to 270.105 kDa, the amino acid lengths extended from 658 to 2441 amino acids, and their isoelectric points varied from 5.49 (IbGLR32) to 9.31 (IbGLR30). Most IbGLRs were stable, only IbGLR2/10/11/13/14/15/21 and IbGLR30 were unstable (instability index > 40). 17 hydrophobic proteins (the grand average of hydropathy [GRAVY] score > 0) and 20 hydrophilic proteins (GRAVY score < 0) were identified for the IbGLR family. Subcellular localization prediction assessment demonstrated that except for IbGLR1 (located on chloroplast) and IbGLR12 (located on chloroplast and nucleus), the remaining IbGLRs were located on the plasma membrane.

**Table 1:** Characterization for *IbGLRs* in sweet potato.

Gene name	Gene ID	CDS (bp)	Phosphorylation site			Amino acids (aa)	MW (kDa)	pI	Instability index	GRAVY	Subcellular locations
			Ser	Thr	Tyr						
*IbGLR1*	g624.t1	2313	13	4	3	770	85.398	5.98	36.00	−0.026	Chloroplast
*IbGLR2*	g641.t1	2778	16	7	3	925	103.146	7.30	46.59	0.008	Plasma membrane
*IbGLR3*	g2074.t1	2790	6	0	4	929	102.309	8.41	31.27	−0.070	Plasma membrane
*IbGLR4*	g10173.t1	2649	8	10	1	882	98.964	6.53	32.61	0.072	Plasma membrane
*IbGLR5*	g10174.t1	2460	21	11	6	819	92.102	8.04	36.39	0.036	Plasma membrane
*IbGLR6*	g10180.t1	2040	7	3	0	679	75.862	6.89	33.80	0.063	Plasma membrane
*IbGLR7*	g10181.t1	1977	5	0	1	658	73.627	6.43	33.86	0.040	Plasma membrane
*IbGLR8*	g16777.t1	2622	13	4	6	873	97.194	6.75	37.85	−0.015	Plasma membrane
*IbGLR9*	g29862.t1	2673	0	0	0	890	99.212	6.59	35.46	0.142	Plasma membrane
*IbGLR10*	g29863.t1	2562	9	13	0	853	95.261	6.30	41.75	0.187	Plasma membrane
*IbGLR11*	g29864.t1	2484	0	0	0	827	91.966	5.97	40.83	0.227	Plasma membrane
*IbGLR12*	g29866.t1	2316	2	2	1	771	86.103	8.25	35.63	0.019	Chloroplast and nucleus
*IbGLR13*	g29867.t1	2823	0	0	0	940	104.895	6.31	41.31	0.021	Plasma membrane
*IbGLR14*	g29868.t1	3507	4	0	3	1168	130.178	8.18	41.71	0.072	Plasma membrane
*IbGLR15*	g29869.t1	2811	13	5	6	936	104.367	5.58	42.27	−0.003	Plasma membrane
*IbGLR16*	g30933.t1	2532	11	5	1	843	93.383	6.15	38.76	−0.012	Plasma membrane
*IbGLR17*	g30934.t1	2922	0	0	0	973	108.564	6.44	33.24	0.010	Plasma membrane
*IbGLR18*	g34576.t1	3129	6	6	0	1042	115.899	7.37	34.06	−0.039	Plasma membrane
*IbGLR19*	g34577.t1	4431	0	0	0	1476	166.657	7.05	38.23	−0.116	Plasma membrane
*IbGLR20*	g37769.t1	2802	0	0	0	933	103.315	5.52	39.79	0.020	Plasma membrane
*IbGLR21*	g37945.t1	2643	0	0	0	880	97.320	6.79	41.35	0.090	Plasma membrane
*IbGLR22*	g49894.t1	3210	7	7	0	1069	119.444	9.08	37.40	−0.088	Plasma membrane
*IbGLR23*	g49895.t1	2331	12	10	2	776	86.988	6.51	34.15	−0.033	Plasma membrane
*IbGLR24*	g49902.t1	2601	22	11	7	866	96.941	7.94	36.89	−0.017	Plasma membrane
*IbGLR25*	g49903.t1	2652	0	1	4	883	98.686	6.12	32.15	−0.009	Plasma membrane
*IbGLR26*	g53987.t1	2652	0	0	0	883	98.219	6.07	34.81	−0.005	Plasma membrane
*IbGLR27*	g54255.t1	2541	2	1	2	846	93.832	8.32	35.25	−0.111	Plasma membrane
*IbGLR28*	g54256.t1	2631	11	13	0	876	97.683	8.88	33.69	−0.101	Plasma membrane
*IbGLR29*	g54698.t1	2802	21	7	6	933	104.088	8.72	32.90	0.059	Plasma membrane
*IbGLR30*	g54783.t1	3960	23	18	4	1319	149.363	9.31	41.56	−0.220	Plasma membrane
*IbGLR31*	g55118.t1	2628	0	0	0	875	97.429	6.44	33.13	0.051	Plasma membrane
*IbGLR32*	g55121.t1	2373	4	3	0	790	87.021	5.49	36.87	−0.018	Plasma membrane
*IbGLR33*	g55122.t1	2844	0	0	0	947	104.829	5.54	33.57	−0.008	Plasma membrane
*IbGLR34*	g55123.t1	2661	0	0	0	886	97.568	5.53	37.38	0.048	Plasma membrane
*IbGLR35*	g55125.t1	2622	0	0	0	873	96.299	8.51	33.15	−0.032	Plasma membrane
*IbGLR36*	g55127.t1	7326	0	0	9	2441	270.105	7.07	34.79	−0.033	Plasma membrane
*IbGLR37*	g60932.t1	2886	0	0	0	961	106.999	5.63	38.12	−0.050	Plasma membrane

CDS, coding sequence; Ser, serine; Thr, threonine; Tyr, tyrosine; MW, molecular weight; pI, isoelectric point.

**Figure 1:**
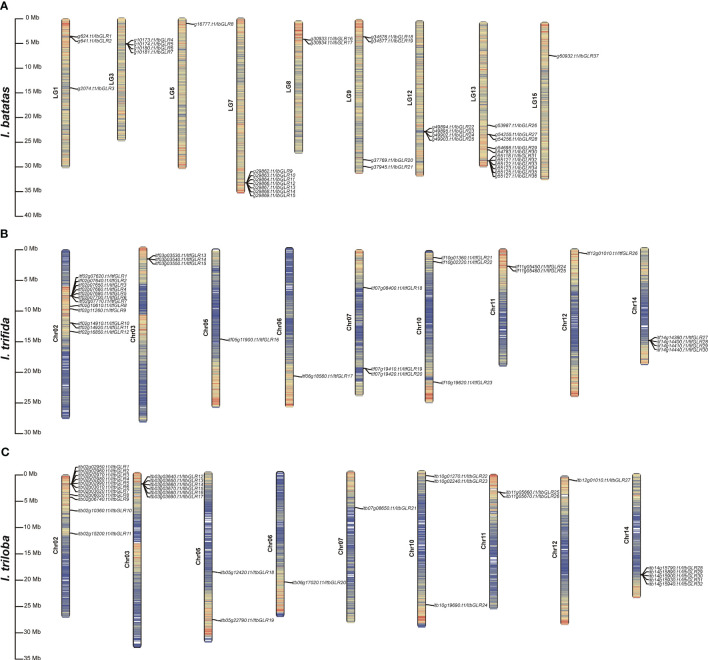
Chromosomal distribution and localization information of *GLR* genes in *Ipomoea batatas* **(A)**, *Ipomoea trifida* **(B)**, and *Ipomoea triloba* **(C)**. The bar chart represents chromosomes, with chromosome numbers on the left side and gene names on the right side for bar chart. The black line on the right side for bar chart marks the position of each *GLR* gene on the chromosome and represents it in units of Mbp.

Based on the physical locations of genes in the *I. batatas*, *I. trifida*, and *I. triloba* genomes, the chromosomal positions of *GLRs* in these crops were mapped. In *I. batatas*, *IbGLR*s were distributed unevenly on nine chromosomes. LG13 had the most *IbGLR* genes, with 11, followed by LG7 with seven. LG3, LG9, and LG12 had four *IbGLRs*. There were less than four *IbGLRs* on LG1, LG5, LG8, and LG15 (**Figure 1A**). In *I. trifida*, except for *ItfGLR31* and *ItfGLR32* located on Chr00 (not shown in **Figure 1B**), the other *ItfGLRs* were located on the same chromosomes (Chr02, Chr03, Chr05, Chr06, Chr07, Chr10, Chr11, Chr12, and Chr14) compared to those in *I. triloba*. Chr02 had the most *ItfGLR* genes, with 12, followed by Chr14 with four. There were less than four *ItfGLRs* on Chr03, Chr05, Chr06, Chr07, Chr10, Chr11, and Chr12 (**Figure 1B**). In *I. triloba*, Chr02 had the most *ItbGLR* genes, with 11, followed by Chr03 with six and Chr14 with five. There were less than four *ItbGLRs* on Chr05, Chr06 Chr07, Chr10, Chr11, and Chr12 (**Figure 1C**). Chr06, Chr10, Chr11, and Chr12 had the same numbers of *GLRs* in *I. trifida* and *I. triloba*. These findings imply that two diploid relatives differed in the proportion and distribution of *GLRs* on chromosomes in comparison to sweet potato.

### 3.2 Phylogenetic relationship of *GLRs* for sweet potato and two diploid relatives

**Figure 2:**
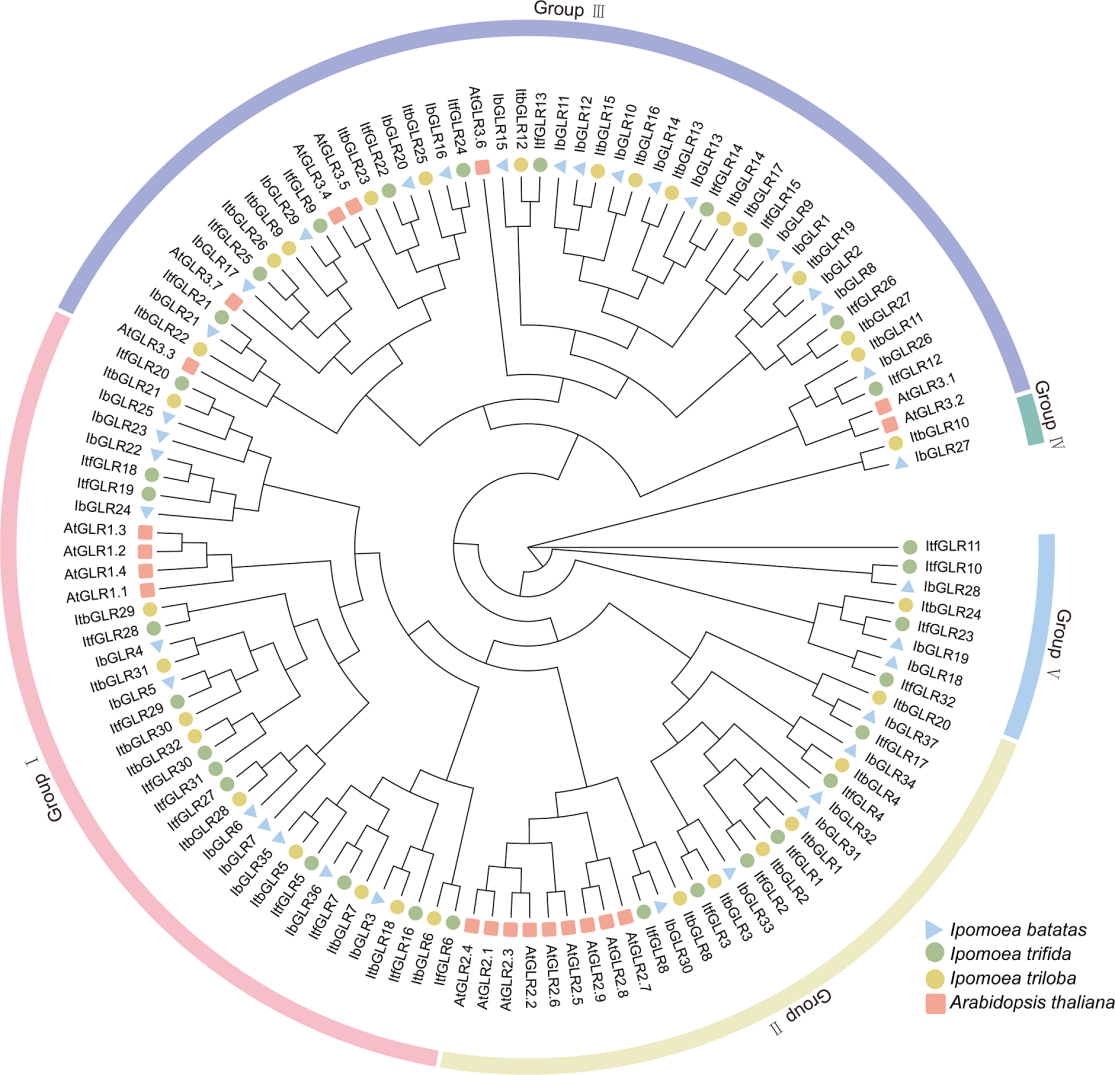
Phylogenetic relationship analysis of GLRs in *Arabidopsis*, *Ipomoea batatas*, *Ipomoea trifida*, and *Ipomoea triloba*. The 20 AtGLRs for *Arabidopsis* are represented by red squares. The 37 IbGLRs for *I.batatas* are represented by blue triangles. The 32 ItfGLRs for *I. trifida* are represented by green circles. The 32 ItbGLRs in *I. triloba* are represented by yellow circles.

Phylogenetic trees were constructed to elucidate the evolutionary interactions between 121 different GLRs from *Arabidopsis* (20), *I. batatas* (37), *I. trifida* (32), and *I. triloba* (32) (**Figure 2**). The GLRs for different species were categorized according to evolutionary distances into different subgroups, including five (Groups I to V) of *I. batatas*, four (Groups I to III, and V) of *I. trifida*, five (Groups I to V) of *I. triloba*, and three (Groups I to III) of *Arabidopsis.* The specific distributions for GLRs were as follows (total: *I. batatas*, *I. trifida*, *I. triloba*, *Arabidopsis*): Group I (37: 11, 12, 10, 4), Group II (27: 6, 6, 6, 9), Group III (47: 16, 10, 14, 7), Group IV (2: 1, 0, 1, 0), and Group V (8: 3, 4, 1, 0) (**Figure 2**; **Supplementary Table S1**). All AtGLRs were found to have homologous proteins in *I. batatas*, *I. trifida*, and *I. triloba*, but IbGLR18/19/27/28, ItfGLR10/11/23/32, and ItbGLR10/24 showed no homology with *Arabidopsis* GLRs. Except for Groups II and IV, the numbers and types of GLRs distributed in the *I. batatas* subgroups were different from those in *I. trifida*, *I. triloba*, and *Arabidopsis*.

### 3.3 GLR conserved motifs and exon–intron structural assessments for sweet potato and two diploid relatives

**Figure 3:**
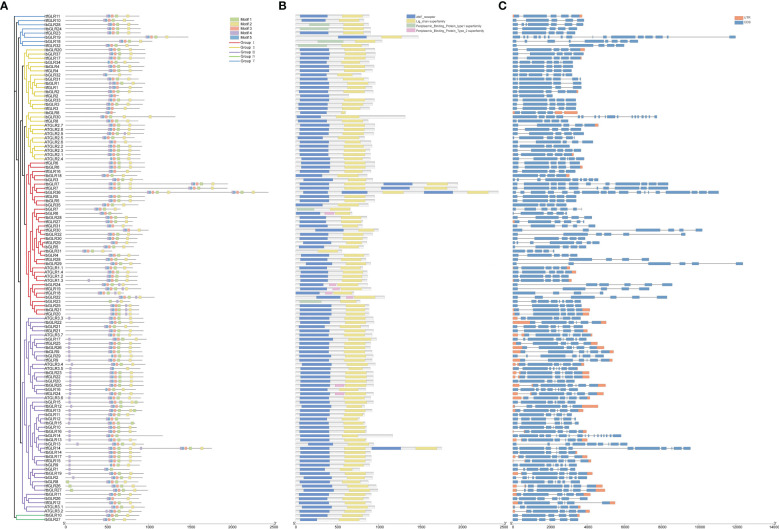
The exon–intron structural and conserved motifs analysis of GLR family in *Ipomoea batatas*, *Ipomoea trifida*, and *Ipomoea triloba.* **(A)** GLRs were distributed in five subgroups by phylogenetic tree (left), and motif 1-5 are shown in different colors (right). **(B)** The GLRs conserved domain structures. The blue boxes, yellow boxes, green boxes, and pink boxes represent the ANF_receptor domain, Lig_chan domain, periplasmic_binding_protein_type1 domain and periplasmic_binding_protein_type2 domain, respectively. **(C)** The GLRs exon-intron structures. The black lines, blue boxes, and orange boxes represent the introns, exons, and UTRs, respectively.

The MEME website was used to analyze the sequence motifs of 37 *IbGLRs*, 32 *ItfGLRs*, and 32 *ItbGLRs*, and motif 1 to 5 were identified as five conserved motifs (**Figure 3A**). All GLR sequences were used to produce the sequences for the five most conserved motifs, shared between sweet potato and two diploid relatives (**Supplementary Figure S1**). Despite being substantially similar, the GLRs for *I. batata*, *I. trifida* together with *I. triloba* could have different numbers and types of conserved domains. For example, ItfGLR1 contained motifs 2 to 5, ItbGLR1 contained motifs 1 to 5, and IbGLR31 contained motifs 1 to 5. ItfGLR4 contained motifs 1 to 5, ItbGLR4 contained motifs 1 to 5, and IbGLR34 contained motifs 1 to 3 and 5. ItfGLR8 comprised motifs 1 to 5, ItbGLR8 contained motifs 3 to 5, and IbGLR30 contained motifs 1 to 5 (**Figure 3A**). Additionally, the receptor domains (atrial natriuretic factor [ANF]_receptor and periplasmic_binding_protein) and four transmembrane helix domains (Lig_chan domains) of GLRs are closely related to plant functions. Most GLRs of *I. batata*, *I. trifida*, and *I. triloba* (30 IbGLRs, 28 ItfGLRs, and 29 ItbGLRs) contained ANF_receptor and Lig_chan domains. However, ItbGLR31 only contained a Lig_chan domain. IbGLR7/18/23/28, ItfGLR32, and ItbGLR24 contained periplasmic_binding_protein_type1 and Lig_chan domains, and IbGLR6/22/24, ItfGLR18/19/24, and ItbGLR25 contained ANF_receptor, periplasmic_binding_protein_type2, and Lig_chan domains (**Figure 3B**).

Exon-intron architectures were examined in order to gain a better understanding of structural variation among the *GLRs* (**Figure 3C**). The number of exons in the GLR genes varied from 3 to 18. In detail, *GLRs* of Group I contained 4 to 18 exons, *GLRs* of Group II contained 3 to 18 exons, *GLRs* of Group III contained 6 to 17 exons, *GLRs* of Group IV contained 5 to 6 exons, and *GLRs* of Group V carried 5 to 17 exons (**Figure 3C**). Additionally, exon-intron architecture for several homologous *GLRs* in *I. batatas* was identified as possibly different from the counterparts in *I. trifida* and *I. triloba*. Such as, *IbGLR36* carried 18 exons, but *ItfGLR7* and *ItbGLR7*, the corresponding homologous genes of *IbGLR36* contained 10 and 11 exons in Group I, *IbGLR30* contained 18 exons, but *ItfGLR8* and *ItbGLR8* both contained five exons in Group II, and *IbGLR13* carried nine exons, but *ItfGLR14* and *ItbGLR14* contained 12 and six exons in Group III (**Figure 3C**). These findings suggest that sweet potato genome potentially underwent a lineage-specific differentiation event involving *GLR* gene family members.

### 3.4 *Cis*-elements assessment for *IbGLR* promoters in sweet potato

**Figure 4:**
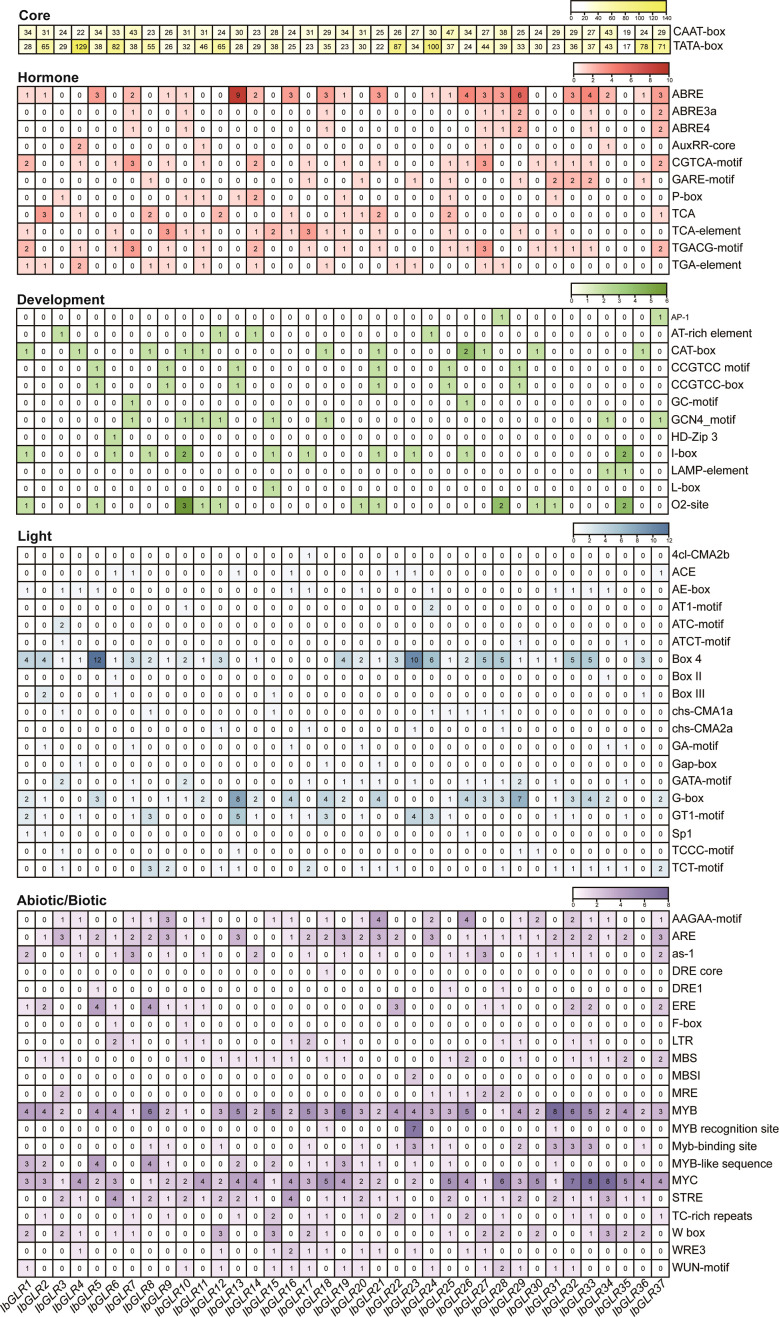
The *cis*-element analysis of *IbGLR*s in *Ipomoea batatas*. Yellow, red, green, blue, and purple represents core elements, hormone elements, development elements, light elements, and abiotic/biotic elements, respectively. For the same color, a darker color indicates a greater number for *cis*-elements.

A *cis*-element, such as the sequence upstream of GLRs, could play a significant role in plant development and stress responses. Hence, we used a 1500 bp DNA sequence upstream of *IbGLRs* for *cis*-element analysis in *I. batatas*. According to the predicted functions, such components were separated into five groups (core, hormone, developmental, light, and abiotic/biotic) (**Figure 4**). The CAAT-box and TATA-box core promoter elements were present in all 37 *IbGLRs*. There were 19 to 47 CAAT-box and 17 to 129 TATA-box core promoter elements in 37 *IbGLRs*. In addition to *IbGLR35*, other *IbGLRs* were found to have several hormone elements, such as the P-box for gibberellic acid (GA)-responsive elements, TCA for SA-responding elements, AuxRR-core together with TGA-element for indole-3-acetic acid (IAA)-responsive elements, CGTCA-motif and TGACG-motif for MeJA-responsive elements/ABRE for ABA-responsive elements (**Figure 4**).

For development-related elements, the CAT-box related to meristem formation (found in *IbGLR1/4/8/10/11/18/21/26/27/30/36*), O2-site, a zein metabolism-regulatory element (observed in *IbGLR1*/*5*/*10*/*11*/*12*/*20*/*21*/*28*/*30*/*31*/*35*), I-box responding to light (found in *IbGLR1*/*6*/*8*/*10*/*15*/*17*/*21*/*23*/*26*/*35*), and GCN4_motif participating in regulating seed-specific expression (found in *IbGLR7*/*10*/*11*/*12*/*15*/*18*/*34*/*37*) were abundant in promoters of *IbGLRs*. The promoter regions of *IbGLR2*/*16*/*19*/*22*/*32*/*33* did not contain any development-related elements (**Figure 4**). Similarly, the promoters of 37 *IbGLRs* contained a number of light-responsive elements. *IbGLRs* were found to be rich in BOX4, G-boxes, GT1-motifs, and TCT-motifs. The ATC-motif was only present in *IbGLR3*, and 4cl-CMA2b only in *IbGLR17* (**Figure 4**).

Moreover, several abiotic elements, the MYC, MYB, and DRE core responded to drought stress, LTR, MBS, and W box responded to salt stress and biotic elements, the WUN, WRE3, and W box motifs were found in most *IbGLRs* (**Figure 4**). This indicated that *IbGLRs* may have different roles under different stress conditions. The DRE core was only found in the promoter region of *IbGLR18*. The results demonstrate that *IbGLRs* take part in regulating the growth and development of plants, hormone crosstalk, and adaptation to abiotic and biotic stress in sweet potato.

### 3.5 Protein interaction networking for IbGLRs within sweet potato

**Figure 5:**
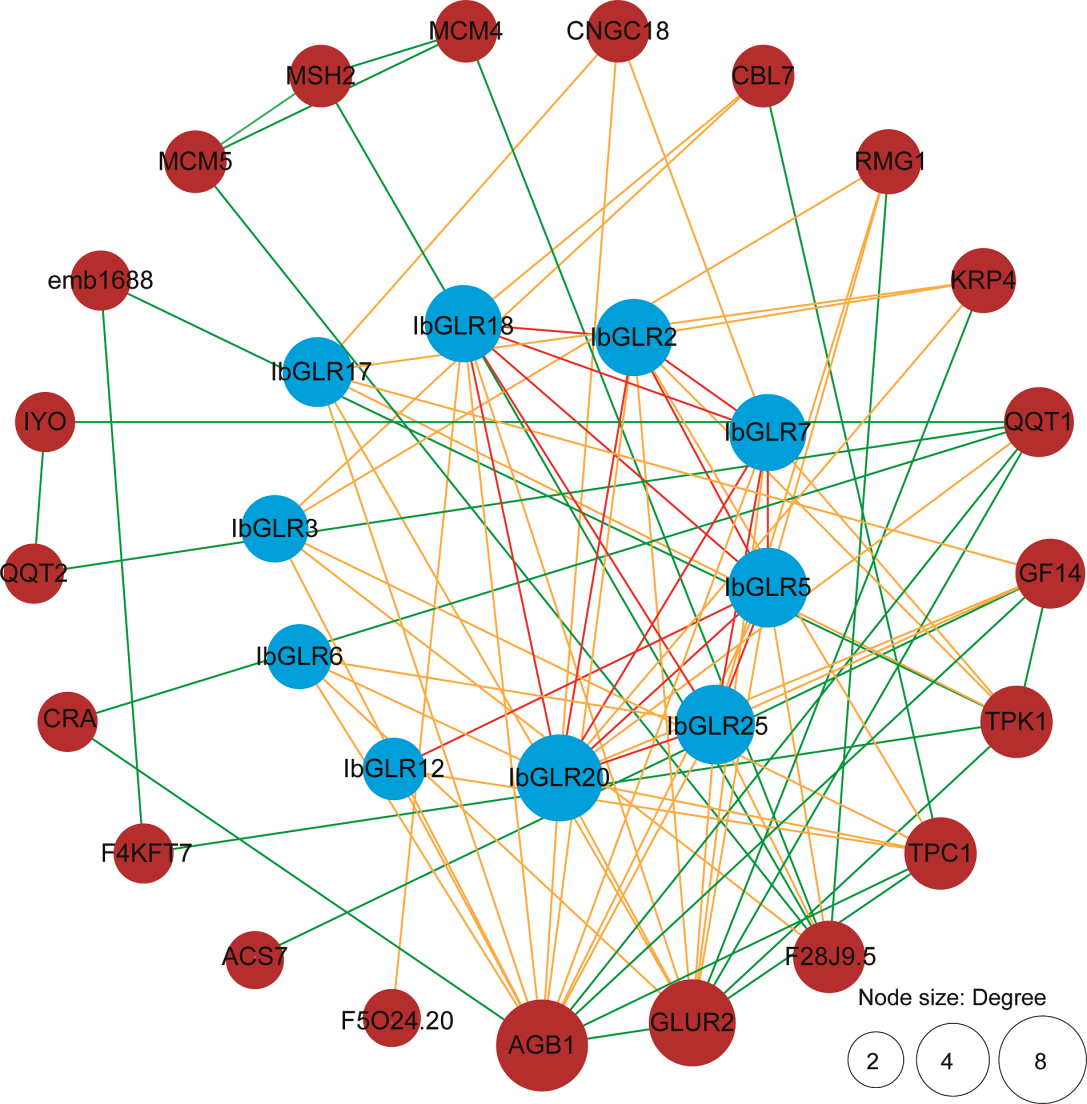
IbGLR functional interaction network analysis in *Ipomoea batatas*. The network nodes represent proteins. The number of interacting proteins is represented by node size. The lines represent the interactions between proteins. The interactions between different GLRs are represented by red lines. The interactions between GLRs and other proteins are represented by orange lines. The interactions between proteins other than GLR are represented by green lines.

An IbGLR interaction network was built through orthologous proteins from *Arabidopsis* to examine possible regulatory networking for IbGLRs (**Figure 5**). The prediction of protein interactions suggested that some IbGLRs (IbGLR2/5/7/12/18/20/25) could interact with other IbGLRs. Moreover, IbGLR7, IbGLR17, and IbGLR20 were determined to interact with CNGC18, which was reported to regulate germination of pollen grains and the growth of pollen tubes (Chang et al., 2007; Cao et al., 2016; Gu et al., 2017). IbGLR17, IbGLR20, and IbGLR25 were determined to interact with GF14, which participates in transport, growth, metabolism, and the drought stress reactions (Li et al., 2016; Hajibarat et al., 2022). IbGLR2, IbGLR7, and IbGLR17 were determined to interact with TPK1, which participates in potassium transportation, fruit ripening, and quality formation (Lu, 2011; Latza et al., 2013; Wang et al., 2018). IbGLR2/3/12/20 were determined to interact with TPC1, which mediates Ca^2+^ release (Moccia et al., 2021; Navarro-Retamal et al., 2021; Pottosin and Dobrovinskaya, 2022), and IbGLR2/3/5/6/7/12/17/18/20 and IbGLR25 were determined to interact with AGB1, which regulates salt stress tolerance, fungal and bacterial immunity, and plant growth (Takahashi et al., 2018; Yu and Assmann, 2018; Zhang et al., 2020; Qi et al., 2021; Afrin et al., 2022). These findings indicate that IbGLRs have a distinct function in mediating biotic and abiotic stress and influence plant development in sweet potatoes.

### 3.6 *GLR* expression for sweet potato and two diploid relatives

#### 3.6.1 Tissue-specific expression assessment

**Figure 6:**
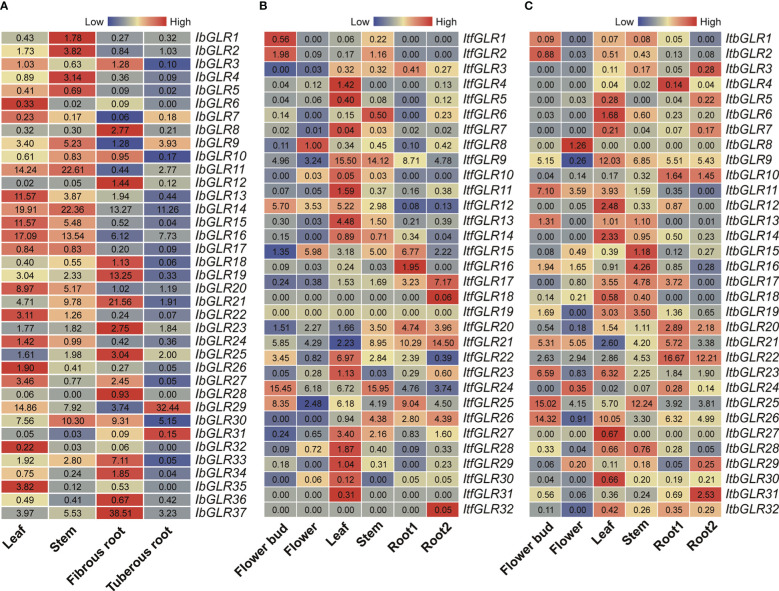
*GLR* expression study in different tissues for different cultivars. **(A)** Represents expression analysis for *Ipomoea batatas* based on the leaf, stem, fibrous root, and tuberous root. **(B, C)** Represent gene expression patterns for *Ipomoea trifida* and *Ipomoea triloba* based on the flower bud, flower, leaf, stem, root 1, and root 2. The FPKM values are displayed in the boxes. The color bar only represents the expression values of one *GLR* gene in different tissues.

*IbGLR* expression levels were examined using RNA-seq data in four typical *I. batatas* tissues (leaves, stems, fibrous roots, and tuberous roots), in order to investigate their potential biological functions in plant developmental processes (**Figure 6A**). Interestingly, different *IbGLR*s had distinct patterns of expression in the four tissues, with some *IbGLRs* showing tissue-specific expression. *IbGLR6/13*/*26*/*32* and *IbGLR35* had a high expression in leaves, whereas the expression of *IbGLR1*/*2* and *IbGLR4* was high in the stems. *IbGLR8*/*12*/*18*/*19*/*21*/*23*/*25*/*28*/*33*/*34*/*36* and *IbGLR37* highly expressed in fibrous roots, *IbGLR29* and *IbGLR31* highly expressed in tuberous roots, *IbGLR3* and *IbGLR27* highly expressed in leaves and fibrous roots, *IbGLR11*/*14*/*15*/*16*/*17*/*20*/*22* and *IbGLR24* highly expressed in leaves and stems, and *IbGLR10* and *IbGLR30* highly expressed in stems and fibrous roots. These findings imply that *IbGLRs* may have different roles in sweet potato tissue development.

Additionally, the expression patterns of *ItfGLRs* and *ItbGLRs* were studied *via* re-analyzing RNA-seq data published in six tissues (flower buds, flowers, leaves, stems, root 1, and root 2) (Wu et al., 2018) (**Figures 6B, C**). In *I. trifida*, *ItfGLR9* was highly expressed in leaves (FPKM value = 15.50) and stems (FPKM value = 14.12). In *I. triloba*, *ItbGLR8*, and *ItbGLR27* were only expressed in flowers and leaves, respectively, whereas the other *ItbGLRs* were expressed in different tissues. *ItbGLR9* exhibited a high expression in leaves. *ItbGLR22* was highly expressed in root 1 and root 2. While *ItbGLR25* was highly expressed in stems and flower buds, *ItbGLR26* exhibited a high expression in leaves and flower buds. These findings suggest that different *GLR* genes exhibit diverse growth regulatory roles in sweet potato and two diploid relatives, as well as different tissue expression patterns.

#### 3.6.2 Expression assessment for *GLRs* during various stages of root development

**Figure 7:**
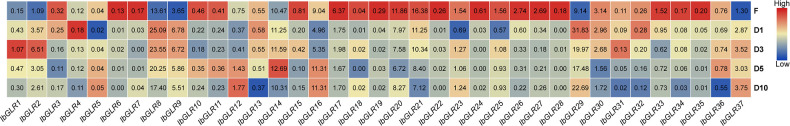
The expression analysis of *IbGLRs* at different stages of root development. F, D1, D3, D5 and D10 represent fibrous root (diameter of approximately 1 mm), initial tuberous root (diameter of approximately 1 cm), tuberous root (diameter of approximately 3 cm), tuberous root (diameter of approximately 5 cm), and tuberous root (diameter of approximately 10 cm), respectively. The FPKM values are displayed in the boxes. The color bar only represents the expression values of one *GLR* gene in different stages of root development.

The expression patterns for *IbGLRs* in the roots of Xushu22 plants were studied at five developmental stages using RNA-seq data (Dong et al., 2019). The results indicated that among the 37 *IbGLRs*, 22 *IbGLRs* shared similar expression patterns, with a higher transcriptional level in fibrous roots (diameter of approximately 1 mm) than roots from other growth stages. Within this group, *IbGLR21* had the highest level of expression (FPKM value = 16.38) (**Figure 7**). The expression of *IbGLR29* was the highest (FPKM value = 31.83) in the initial tuberous root (diameter of approximately 1 cm), and the expression of *IbGLR2* was the highest (FPKM value = 6.51) in tuberous roots (diameter of approximately 3 cm). Meanwhile, the expression of *IbGLR14* was highest (FPKM value = 12.69) in tuberous roots (diameter of approximately 5 cm), and the expression of *IbGLR16* was the highest (FPKM value = 11.31) in tuberous roots (diameter of approximately 10 cm), which was consistent with the tuberous root of approximately 5 cm in diameter. Overall, the expression patterns imply different contributions of *IbGLRs* in the root development of sweet potato.

#### 3.6.3 Expression assessment for *GLRs* in resistant and susceptible varieties under root rot treatment conditions

**Figure 8:**
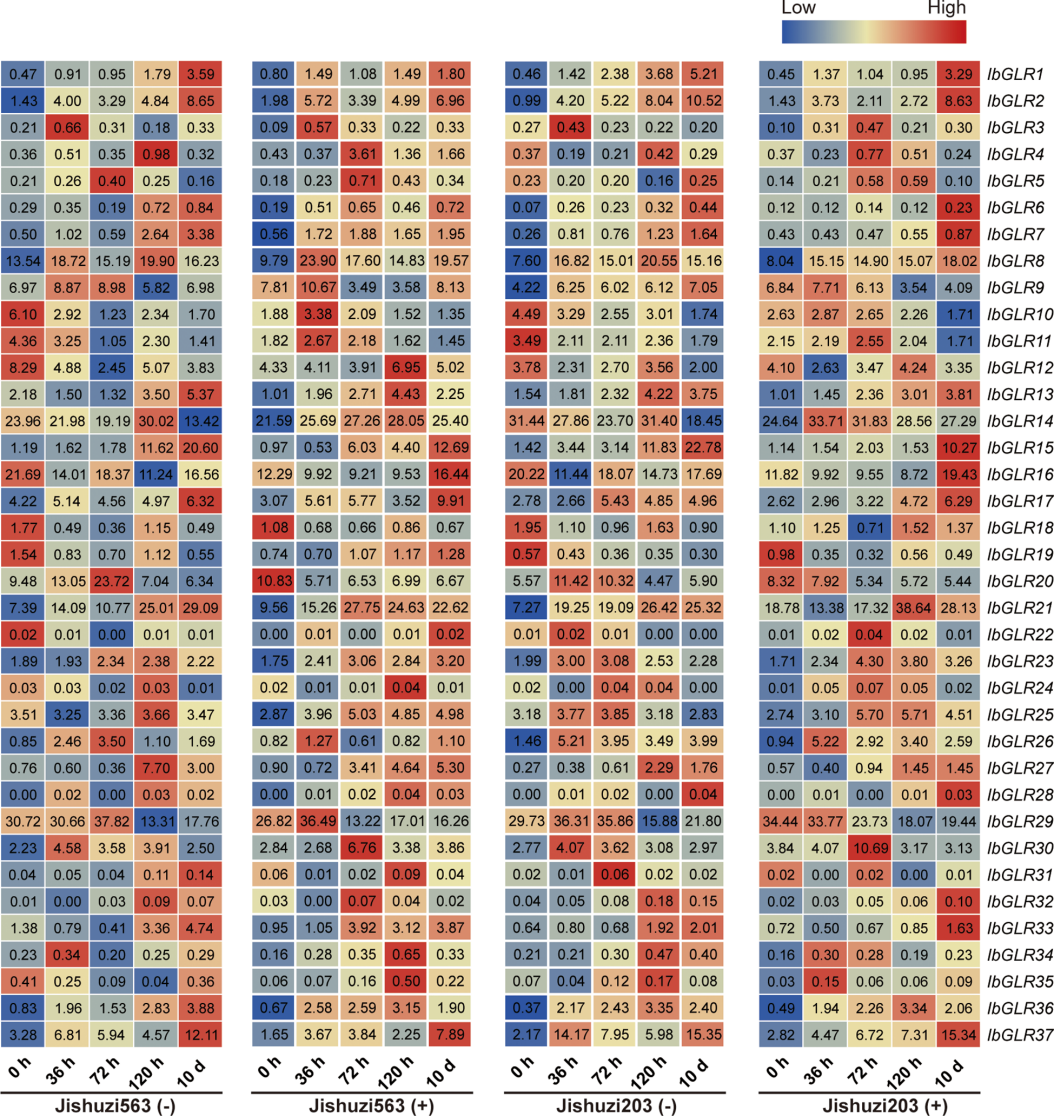
*IbGLRs* expression analysis in response to root rot. Jishuzi563(-): a root rot sensitive variety without root rot; Jishuzi563(+): a root rot sensitive variety with root rot; Jishuzi203(-): a root rot resistant variety without root rot; Jishuzi203(+): a root rot resistant variety with root rot. The FPKM values are displayed in the boxes. The color bar only represents the expression values of one *GLR* gene at different times after root rot induction in different varieties.

To study the possible role of *IbGLRs* during interactions with the sweet potato root rot pathogen, we analyzed the expression levels of *IbGLRs* at different time points after root rot induction in Jishuzi563 (root rot-sensitive variety) and Jishuzi203 (root rot-resistant variety) using RNA-seq data (**Figure 8**). Subsequently, we further evaluated the expression levels of some *IbGLRs* using qRT-PCR (**Supplementary Figure S2**). Without root rot infection, the expression of *IbGLR10*/*11*/*12*/*16*/*18* was repressed at 36 h, 72 h, 120 h, and 10 d compared with that at 0 h in Jishuzi563 and Jishuzi203. After root rot infection, only *IbGLR18* expression was repressed in Jishuzi563 infected by the root rot but was induced in Jishuzi203 infected by the root rot. This result was consistent with qRT-PCR results. It has been speculated that *IbGLR18* may participate in sweet potato resistance to root rot. In addition, the RNA-seq results showed that *IbGLR14/26*/*37* expression levels were upregulated in Jishuzi563 and Jishuzi203 in response to root rot, and the expression levels in the resistant variety Jishuzi203 were higher than those in susceptible variety Jishuzi563, which was consistent with real-time quantitative results. qRT-PCR results showed that *IbGLR14* manifested the highest level of expression at 120 h, *IbGLR26* at 72 h, and *IbGLR37* at 10 d. The outcome suggests that different *IbGLRs* may function differently in developing disease resistance at different stages during the interaction between sweet potato and root rot pathogens.

#### 3.6.4 Expression assessment for *GLRs* in response to salt and drought stresses in hexaploid sweet potato and two diploid relatives

**Figure 9:**
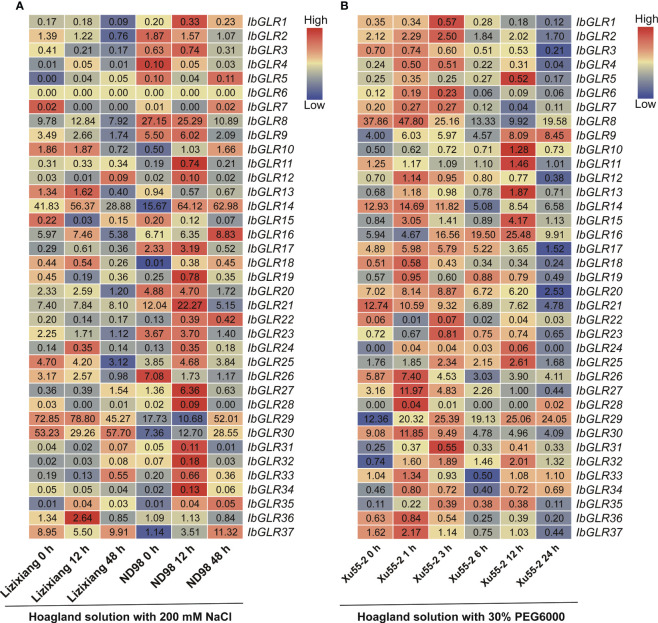
*IbGLRs* expression analysis in response to NaCl and PEG treatments. **(A)** Expression analysis for *IbGLRs* under NaCl treatment conditions in salt-sensitive variety Lizixiang and salt-tolerant line ND98. **(B)** Expression analysis for *IbGLRs* under PEG treatment conditions in drought-tolerant variety Xu55-2. The FPKM values are displayed in the boxes. The color bar only represents the expression values of one *IbGLR* in different treatments.

To better understand the capacity of *IbGLRs* to combat abiotic stress, RNA-seq data from a salt-tolerant line (ND98) and a salt-sensitive variety (Lizixiang) were used to compare the expression patterns of *IbGLRs* under salt stress. Meanwhile, the RNA-seq data from Xu55-2, a drought-resistant cultivar, was studied under drought stress (Zhang et al., 2017; Zhu et al., 2019). Salt stress enhanced the expression of 13 *IbGLRs* in the ND98 line (the expression for *IbGLR1/11/14/19/24/33/34* was highest at 12 h, while that of *IbGLR10/18/22/30/35/37* was highest at 48 h). For these genes, *IbGLR19* expression in salt sensitive variety Lizixiang was repressed by salt stress (**Figure 9A**). In Xu55-2, the expression of 26 *IbGLRs* was induced rapidly following drought stress and reached its maximum at the early stage (≤3 h). Meanwhile, *IbGLR21* expression was repressed by polyethylene glycol (PEG) treatment, whereas *IbGLR9/10/13/15/19/29/31/32* expression levels were induced (**Figure 9B**). Thus, it may be speculated that *IbGLR19* is related to salt and drought tolerance in sweet potato.

**Figure 10:**
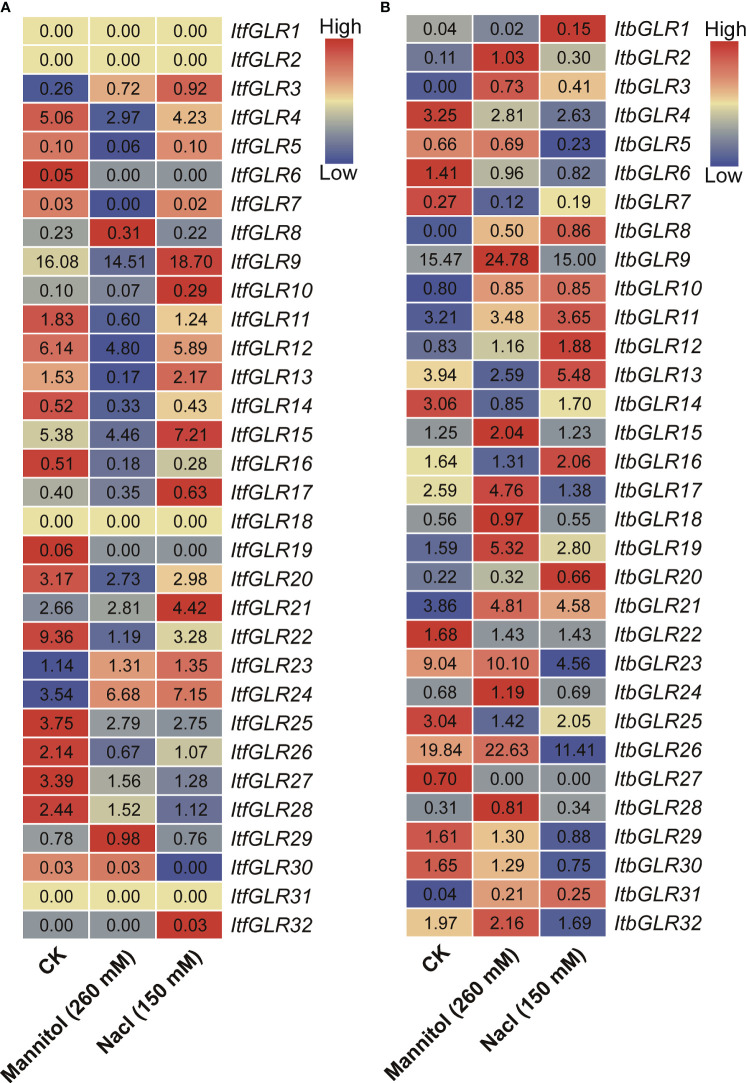
*GLRs* expression analysis in response to mannitol and NaCl stresses in *Ipomoea trifida* **(A)** and *Ipomoea triloba* **(B)**. The FPKM values are displayed in the boxes. The color bar only represents the expression values of one *GLR* gene in different treatments.

Additionally, the evaluation of *ItfGLRs* and *ItbGLRs* expression in *I. trifida* and *I. triloba* following drought and salt treatment was conducted using RNA-seq data (Wu et al., 2018). Expression levels of *ItfGLR1*/*2*/*18*/*31* were not induced, whereas those for *ItfGLR3*/*23*/*24* and *ItbGLR3*/*8*/*10*/*11*/*21*/*31* were induced (**Figure 10**). These results indicate that there are differences in the expression of *GLRs* in sweet potato and two diploid relatives under drought and salt stress.

#### 3.6.5 Comparative investigation of *ItfGLRs* and *ItbGLRs* expression in response to hormone and temperature stress

**Figure 11:**
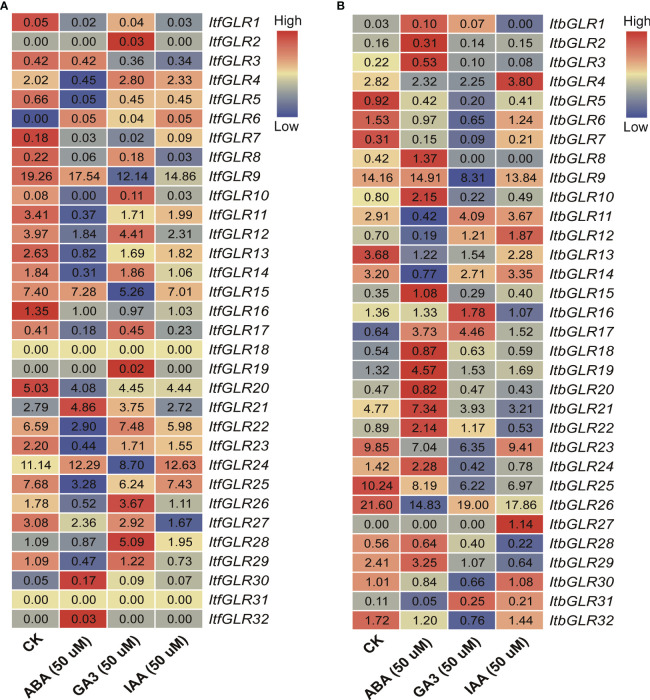
*GLRs* expression analysis in response to abscisic acid (ABA), gibberellin (GA), and indolent-3-acetic acid (IAA) treatments in *Ipomoea trifida* **(A)** and *Ipomoea triloba* **(B)**. The FPKM values are displayed in the boxes. The color bar only represents the expression values of one *GLR* gene in different treatments.

Ultimately, an assessment of expression patterns for *ItfGLRs* and *ItbGLRs* under ABA, GA, and IAA treatments was carried out through RNA-seq data from *I. trifida* and *I. triloba* (Wu et al., 2018). In *I. trifida*, the expression of *ItfGLR18* and *ItfGLR31* was not induced by any of the hormones, whereas the expression levels of five *ItfGLRs* were induced by ABA, those of 11 *ItfGLRs* were induced by GA, and those of two *ItfGLRs* were induced by IAA. *ItfGLR6* and *ItfGLR30* expression was induced by all hormones, and *ItfGLR1/5/7/8/9/11/13/15/16/20/23/25* and *ItfGLR27* expression was suppressed by the three hormones (**Figure 11A**). In the case of *I. triloba*, expression levels of 15 *ItbGLRs* were induced by ABA, those of four *ItbGLRs* were induced by GA, and those of five *ItbGLRs* were induced by IAA. *ItbGLR18* and *ItbGLR19* expression was induced by all hormones, whereas *ItbGLR5/6/7/13/23/25/26* and *ItbGLR32* expression was repressed by all hormones (**Figure 11B**). The findings suggest that *GLRs* in the two diploid relatives participate in various hormonal pathways and take part in the crosstalk among different hormones.

**Figure 12:**
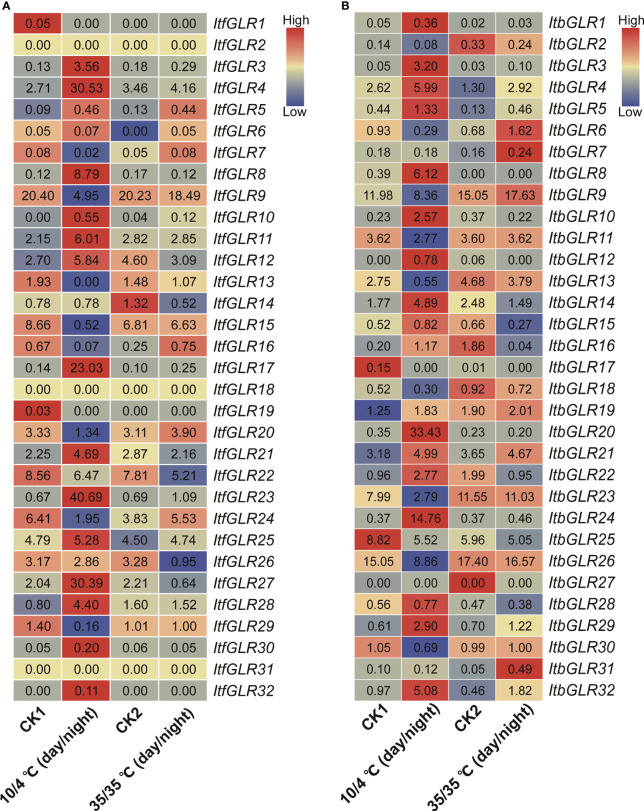
*GLRs* expression analysis under 10/4°C (day/night) and 35/35°C (day/night) treatments in *Ipomoea trifida* **(A)** and *Ipomoea triloba* **(B)**. CK1, cold control; CK2, heat control. FPKM values are shown in the boxes. The color bar only represents the expression values of one *GLR* gene in different treatments.

Additionally, the pattern of expression for *ItfGLRs* and *ItbGLRs* was analyzed utilizing *I. trifida* and *I. triloba* RNA-seq data, respectively, at 10/4°C (day/night) and 35/35°C (day/night) treatment (Wu et al., 2018). In *I. trifida*, *ItfGLR4/8*/*17/23*/*27* and *ItfGLR28* expression was induced by 10/4°C (day/night), whereas *ItfGLR16* expression was induced by 35/35°C (day/night) temperatures comparison with the control levels (**Figure 12A**). In *I. triloba*, *ItbGLR3*/*8/20*/*24*/*29* and *ItbGLR32* expression was induced by 10/4°C (day/night) and *ItfGLR31* expression was induced by 35/35°C (day/night) compared with control levels temperatures (**Figure 12B**). These results show that different *GLRs* participate in the responses of *I. trifida* and *I. triloba* to different temperature stresses.

## 4 Discussion

Plant GLRs are mainly located on vacuolar and plasma membranes (Davenport, 2002). They are potential candidates for the plasma membrane-level regulation for calcium influx (Mäser et al., 2001) and could be amino acid sensors in plants (Tapken et al., 2013). *Arabidopsis*, rice, and other model plants have been investigated for the functions of *GLRs*, however, until now, there have been no reports on the identification of the *GLR* gene family members in sweet potato. Because of the complicated genetic background of cultivated sweet potato, research on the sweet potato gene family is limited in the cultivated variety, mostly focusing on its two diploid relatives (Chen et al., 2019a; Chen et al., 2019b; Li et al., 2019; Lu et al., 2019; Wan et al., 2020; Zhu et al., 2020; Huang et al., 2021; Dai et al., 2022; Nie et al., 2023). Through this investigation, we conducted an in-depth study of the characteristics of *GLR* genes and compared their expression patterns in response to different types of biotic and abiotic stresses using genomic sequences for the hexaploid sweet potato and two diploid relatives. The genome-wide study of *GLR* genes provides vital guidance for the further studying their functions.

### 4.1 Evolution of *GLR* genes for sweet potato and two diploid relatives

Overall, *I. trifida* and *I. triloba* had the same number of *GLRs* (32 *ItfGLRs* and 32 *ItbGLRs*) identified, however they were less numerous than those (37 *IbGLRs*) in *I. batatas.* This is consistent with the fact that *I. trifida* and *I. triloba* are diploid and have a close genetic relationship. The evolution and differentiation of chromosomes were revealed by genomic alignment (Mukherjee et al., 2018). *I. batatas, I. trifida*, and *I. triloba* differed in terms of the distribution and proportion of *GLRs* in individual chromosomes. *GLRs* were located on nine chromosomes for *I. batatas* and *I. triloba*, however, *GLRs* were located on ten chromosomes for *I. trifida*. If different members of the same family are located within the same or adjacent intergenic regions, they have tandem repeat relationships (Freeling, 2009). Based on this standard, eight, six, and four tandem repeats were identified in *I. batatas*, *I. trifida*, and *I. triloba*, respectively (**Figure 1**). This indicates that tandem repeats might be one of the reasons for the amplification of *GLR* genes in *I. batatas*, *I. trifida*, and *I. triloba*.

This research is based on the GLRs for *I. batatas, I. trifida*, *and I. triloba*, which were segregated into five groups (Groups I to V), four groups (Groups I to III and V), and five groups (Groups I to V), respectively. The fourth and fifth subgroups consisted of 10 GLRs that were absent in *Arabidopsis*. The type and number of GLRs in various subgroups for sweet potato and two diploid relatives are different from those in *Arabidopsis*. These findings imply that the genomes may have experienced lineage-specific differentiation for *GLR* gene family.

Introns are important components of eukaryotic protein-coding genes, eliminated during messenger RNA precursor molecule splicing. They are characterized by their clear organization and abundance in eukaryotic genes (Rogozin et al., 2005; Mukherjee et al., 2018). Because of the presence of introns, gene expression in eukaryotic cells is much more complex than that in prokaryotic cells. Under stressful conditions, introns assume a key role in regulating cell growth (Morgan et al., 2019). Herein, in comparison with *I. trifida* and *I. triloba*, *I. batatas* had distinct exon-intron patterns for some homologous *GLRs* (**Figure 3C**). For example, *IbGLR36*, which was expressed in fibrous roots, contained 17 introns, whereas its homologous genes *ItfGLR7* and *ItbGLR7* contained nine and ten introns, respectively, and were expressed in leaves. Moreover, *IbGLR30*, which was expressed in the stem, contained 17 introns, whereas *ItfGLR8* and *ItbGLR8* contained four and five introns, respectively, and were expressed in flowers (**Figures 3C**, **6**). The corresponding differences in exon–intron structure between sweet potato and its two diploid relatives might lead to different functions for *GLRs* in various aspects of plant growth and development (Pang et al., 2018; Ma et al., 2019; Ma J et al., 2020).

### 4.2 Hormone crosstalk roles of *GLRs* for sweet potato and two diploid relatives

Plant *GLRs* are actively involved in hormone biosynthesis and signal transduction concerning the regulation of plant growth and responses to stress (Weiland et al., 2015). *PpGLR1* is involved in ABA-mediated growth regulation in *Physcomitrium patens* (Wang et al., 2022). Meanwhile, *RsGluR* could serve as a defensive mechanism against pathogen infection by triggering MeJA biosynthesis (Kang et al., 2006). In this work, the promoters of the *IbGLRs* contained 11 hormone-responsive elements, 12 developmental elements, 19 light-responsive elements, and 21 abiotic/biotic-response elements. In addition to *IbGLR35*, other *IbGLRs* comprised at least one hormone element (**Figure 4**). *IbGLR3* only contained the P-box of the GA-responsive elements, whereas its homologous gene *ItbGLR18* was determined to be induced by ABA. *IbGLR4* was found to contain the TGACG-motif and CGTCA-motif for MeJA-responsive elements, TCA for SA-responsive elements, and the AuxRR-core together with TGA-element for IAA-responsive elements, whereas its homologous gene, *ItbGLR31*, was determined to be induced by GA and IAA. *IbGLR21* contained ABRE for ABA-responsive elements, the TGACG-motif together with CGTCA-motif for MeJA-responsive elements, and TCA for SA-responsive elements, whereas its homologous genes, *ItfGLR21* and *ItbGLR22*, were both determined to be induced by ABA and GA. *IbGLR30* carried the TGACG-motif and CGTCA-motif for MeJA-responsive elements, whereas its homologous gene, *ItbGLR8*, was determined to be induced by ABA (**Figures 4**, **11**). Such results demonstrate *GLRs* involvement in the interplay of several hormones and the participation of homologous *GLR* genes in numerous hormone pathways in sweet potato and two diploid relatives. Further research is needed to fully understand how *GLRs* control hormonal crosstalk.

### 4.3 Functions of *GLRs* in biotic stress responses and root growth of sweet potato and two diploid relatives

*GLR* plays an indispensable role in the response of plants to different biological stresses (He et al., 2016). Liu and colleagues discovered a point mutation for *GhGLR4.8* exon that can increase the resistance of upland cotton to *Fusarium* wilt, while knocking out the *GhGLR4.8* lead the defense ability of cotton cell wall against *G. hirsutum Fov race* 7 to weaken (Liu et al., 2021). *AtGLR3.3* also has a valuable function in the defense response induced by *Pseudomonas syringae* pv *tomato* DC3000. An *AtGLR3.3* mutant was shown to exhibit high sensitivity to DC3000, and the response of defense genes was reduced in mutant lines induced by pathogenic bacteria (Li et al., 2013). Sweet potato root rot infection usually begins at the underground stem and tip or middle of fibrous roots. Severely diseased plants do not produce tuberous roots, whereas slightly diseased plants produce tuberous roots with diseased spots (Ma Z et al., 2020).

Transcriptome and qRT-PCR analysis showed that *IbGLR18* expression was induced in Jishuzi203 (root rot-resistant variety) and repressed in Jishuzi563 (root rot-sensitive variety) after root rot infection. In addition, the *IbGLR18* expression in fibrous roots was higher in comparison to that in other tissues (leaf, stem, and tuberous root). Furthermore, *IbGLR18* expression was also higher in the fibrous root (diameter of root 1 mm) than that in tuberous roots (diameter of root 1/3/5/10 cm) in the five developmental stages of the Xushu22 root. Therefore, we speculate that *IbGLR18* might be involved in resistance to root rot and the formation of fibrous roots in sweet potato, but its function should be verified in the future studies.

Usually, tuberous roots are the main harvesting tissues for hexaploid sweet potato. *I*. *trifida* and *I. triloba* are not capable of forming tuberous roots (Wu et al., 2018). The expression for *IbGLR29* in tuberous roots was higher than that in other tissues (leaf, stem, and fibrous roots) of sweet potato, and its homolog genes *ItfGLR9* and *ItbGLR9* were highly expressed in the leaf. Further, in the five developmental stages of Xushu22 roots, the expression of *IbGLR29* in tuberous roots (root diameter of 1/3/5/10 cm) was high in comparison to that in fibrous roots (diameter of approximately 1 mm). *IbGLR29* may therefore be involved in the generation of tuberous roots in sweet potato.

### 4.4 Functions of *GLRs* in abiotic stress response of sweet potato and two diploid relatives

*GLRs* play a crucial role in mediating plants responses to abiotic environmental stress (He et al., 2016). The degree of expression of *ZmGLR2.3/3.1* demonstrated a significant increase after drought stress (Zhou et al., 2021). In this work, MYB and MYC were found to respond to drought stress, MBS to salt stress, and LTR to cryogenic stress, and these were identified in the *IbGLR19* promoter, which was rapidly expressed after drought stress and reached its expression maximum at the early stage (1 h). Moreover, its expression was upregulated by NaCl treatment in ND98 (salt-tolerant line) and downregulated in Lizixiang (salt-sensitive variety, **Figures 4**, **9**). *ItfGLR23*, the homolog gene of *IbGLR19*, was induced by both drought and salt treatments in *I. trifida* (**Figure 10**). These results suggest that *IbGLR19* may contribute to abiotic stress. Additionally, *I. trifida* and *I*. *triloba* may be utilized for searching and identifying functional genes, particularly those that provide tolerance or resistance to biotic and abiotic stresses, which may have been lost during the domestication of cultivated sweet potato (Cao et al., 2016). In *I. trifida* and *I. triloba*, several genes (*ItfGLR8* and its homologous gene *ItbGLR8*, *ItfGLR17* and its homologous gene *ItbGLR20*, *ItfGLR23* and its homologous gene *ItbGLR24*, and *ItfGLR28* and its homologous gene *ItbGLR29*) exhibited the same expression patterns and were induced under 10/4°C (day/night) (**Figure 12**). *ItfGLR3* and its homologous gene *ItbGLR3* were induced by drought and salt treatments (**Figure 10**). In summary, the *GLRs* induced in *I. trifida* and *I. triloba* could serve as candidate genes for enhancing the abiotic stress resistance of sweet potato.

## Data availability statement

The original contributions presented in the study are publicly available. This data can be found here: NCBI SRA (http://www.ncbi.nlm.nih.gov/Traces/sra), accession numbers SAMN10755180, SAMN10755181, SAMN10755182, SAMN10755183, SAMN10755184, SAMN10755185, SAMN10755186, SAMN10755187, SAMN10755188, SAMN10755189, SAMN10755190, SAMN10755191, SAMN10755192, SAMN10755193, SAMN10755194 and SRP092215.

## Author contributions

YH: Writing – original draft, Writing – review & editing. ZD: Writing – original draft. JH: Writing – original draft. MH: Writing – original draft. ZW: Writing – original draft. WJ: Writing – original draft. ZG: Writing – original draft. XL: Writing – review & editing. LL: Writing – review & editing. ZM: Writing – review & editing.
