# Supplementary figures and images for: Genome-wide identification and expression analysis of the glutamate receptor gene family in sweet potato and its two diploid relatives
Source: Front Plant Sci. 2023 Dec 21;14:1255805. doi: 10.3389/fpls.2023.1255805 (PMC10764598; doi:10.3389/fpls.2023.1255805)

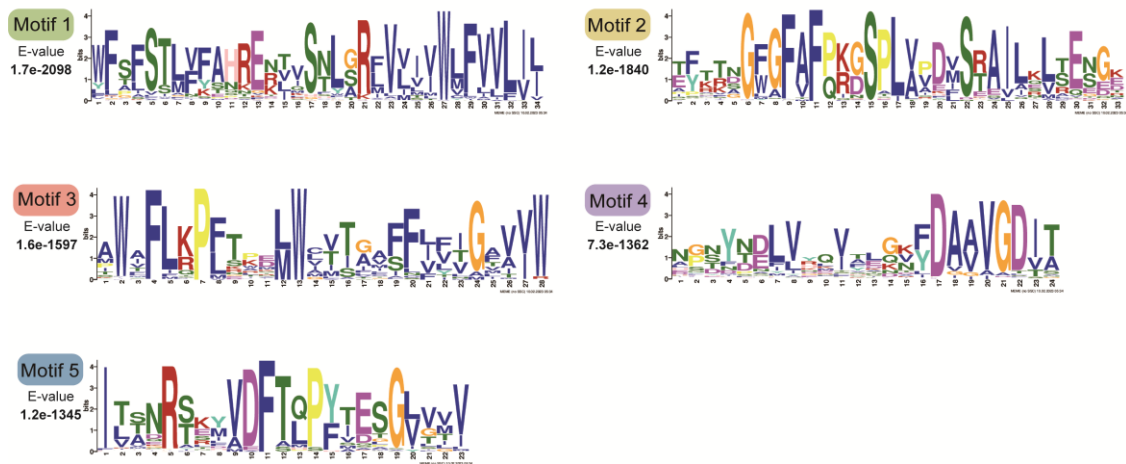

**Figure S1.** Conserved motifs analysis of IbGLRs in *Ipomoea batatas*.

Supplement: Supplementary file 3 [file Image_1.pdf]
