# Supplementary material for: Genome-wide identification and expression analysis of the glutamate receptor gene family in sweet potato and its two diploid relatives
Source: Front Plant Sci. 2023 Dec 21;14:1255805. doi: 10.3389/fpls.2023.1255805 (PMC10764598; doi:10.3389/fpls.2023.1255805)
Supplement: Supplementary file 4 [file Image_2.pdf]

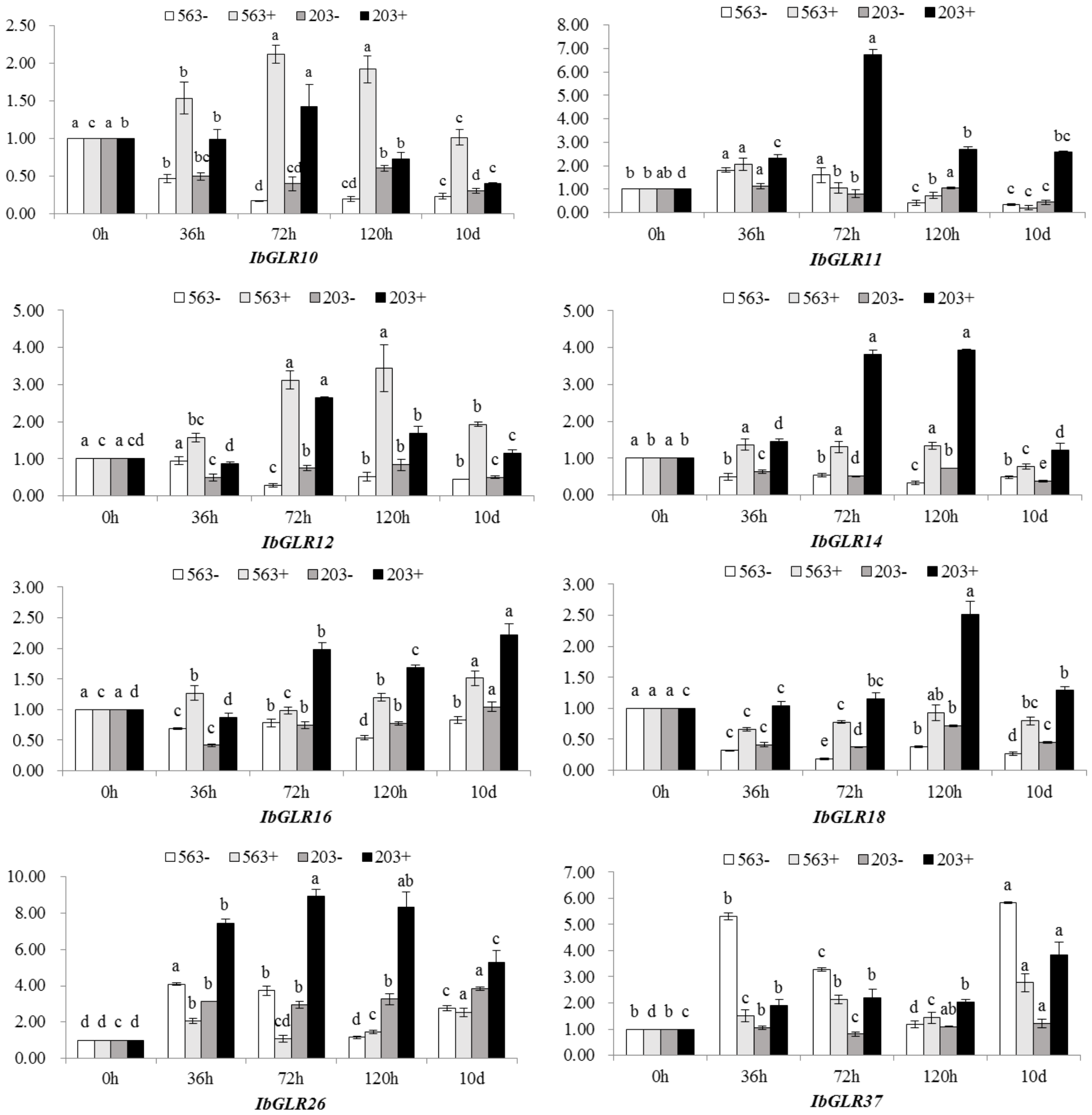


**Figure S2.** The expression analysis for *IbGLRs* in response to root rot. The values were determined by qRT-PCR from three biological replicates consisting of pools of three plants, and the results were analyzed using the $2^{-\Delta ct}$ method, and data were analyzed with DUNCAN's multiple range test ($p < 0.05$). 563-: Jishuzi563 without root rot; 563+: Jishuzi563 with root rot; 203-: Jishuzi203 without root rot; 203+: Jishuzi203 with root rot.
